# Supplementary material for: Single-cell analysis of human primary prostate cancer reveals the heterogeneity of tumor-associated epithelial cell states
Source: Nat Commun. 2022 Jan 10;13:141. doi: 10.1038/s41467-021-27322-4 (PMC8748675; doi:10.1038/s41467-021-27322-4)
Supplement: Supplementary file 1 — Supplemental Information [file 41467_2021_27322_MOESM1_ESM.pdf]

Supplementary Information

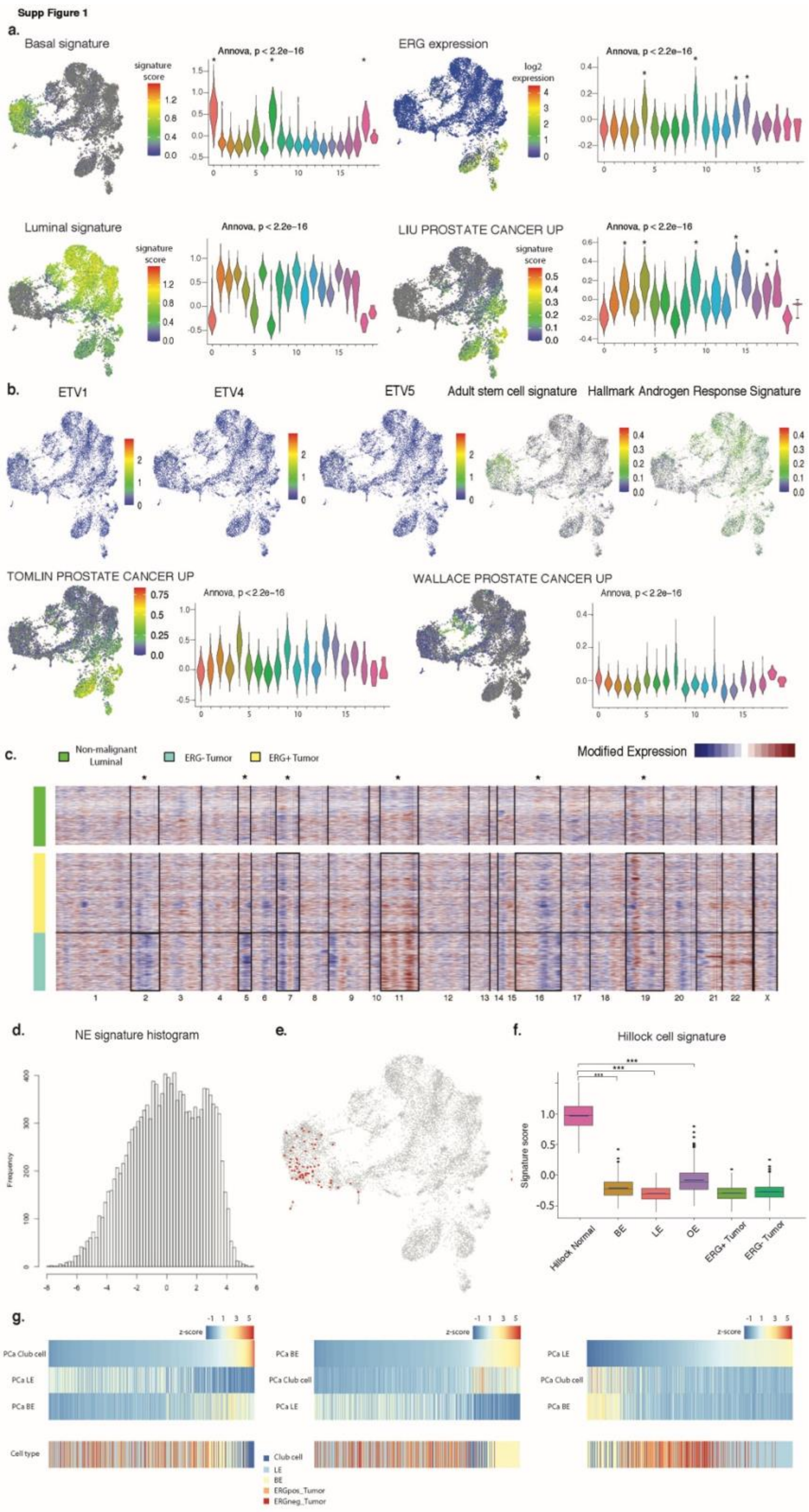

Supplementary Figure 1. Epithelial cell type annotation and validation. a. Left, Uniform Manifold Approximation and Projection (UMAP) projection of normal basal epithelial (BE), luminal epithelial (LE) cell signatures, *ERG* expression and LIU PROSTATE CANCER UP signature, with corresponding violin plots for epithelial cell clusters (*p*-values of one-way ANOVA test are labeled, clusters with upregulated signature scores are highlighted with asterisks). **b.** Top, Featureplots of *ETV1, ETV4, ETV5* expression, adult stem cell signature score, and Hallmark androgen response signature score. Bottom, UMAPs of Tomlin prostate cancer up and Wallace prostate cancer up tumor marker gene sets signature scores with corresponding violin plots. **c.** InferCNV result with significant copy number variations highlighted. **d.** Distribution of neuroendocrine (NE) signature score. **e.** UMAP of epithelial cells with identified NE highlighted in red. **f.** Hillock cell signature score comparison between prostate cancer (PCa) epithelial cells and hillock cells from normal samples (N = 13,322 PCa epithelial cells + 2,530 normal hillock cells, ***: $p < 0.001$, Wilcoxon rank sum test; hillock normal and BE: p = 3.69e-292; hillock normal and LE: p < 2.2e-16; hillock normal and OE: p = 1.70e-216). Center, bounds and percentiles are shown in the box plot. **g.** Single-set Gene Set Enrichment Analysis (ssGSEA) validation of PCa BE, LE, and club cell signature gene sets. z-score of ssGSEA signature scores are computed and ordered. Source data are provided as a Source Data file.

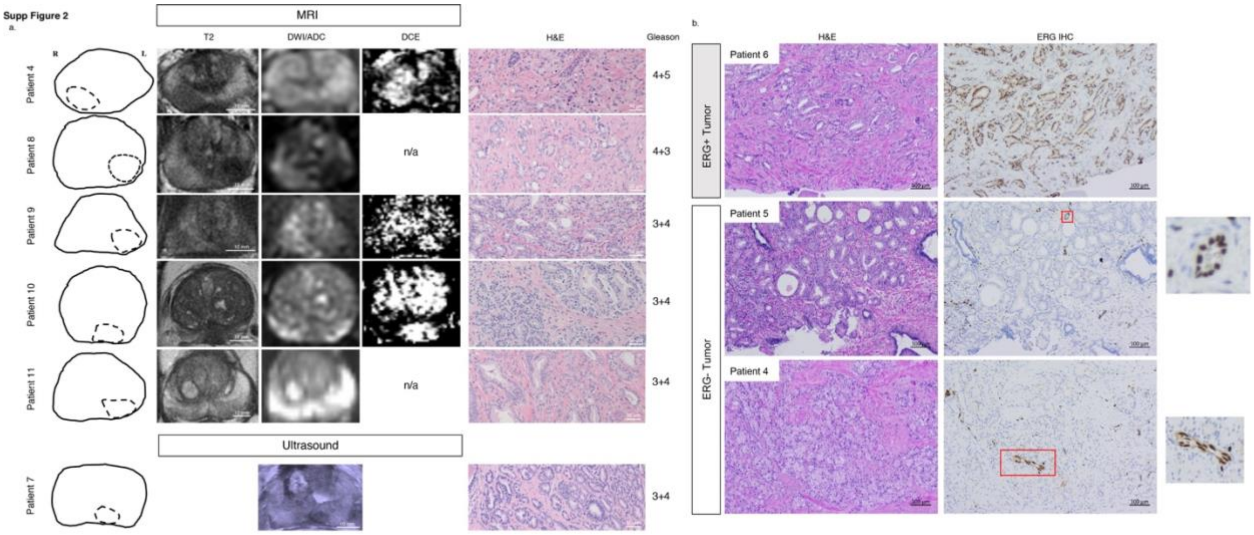

Supplementary Figure 2. Histopathological assessment of tumor cells and ERG staining confirmed tumor content. a. Lesions that were sampled from radical prostatectomy specimens corresponded to PI-RADSv2 4 and 5 MRI lesions or hypoechoic ultrasound lesions. Representative H&E images of these lesions along with Gleason grade are shown. DWI/ADC: Diffusion-weighted imaging / apparent diffusion coefficient. DCE: Dynamic contrast enhanced imaging. H&E: Hematoxylin and Eosin stain. b. Serial sections of primary tumors from Patient 4,5, and 6 were stained with H&E and erg immunohistochemistry. Endothelial cells in *ERG*-negative (*ERG*-) tumors (inset) were magnified. Histopathological assessment of tumor cells were conducted for a total of eight patients and *ERG* staining confirmation were conducted only for the three patients due to the limitation of sample quality of the patient tumor tissues.

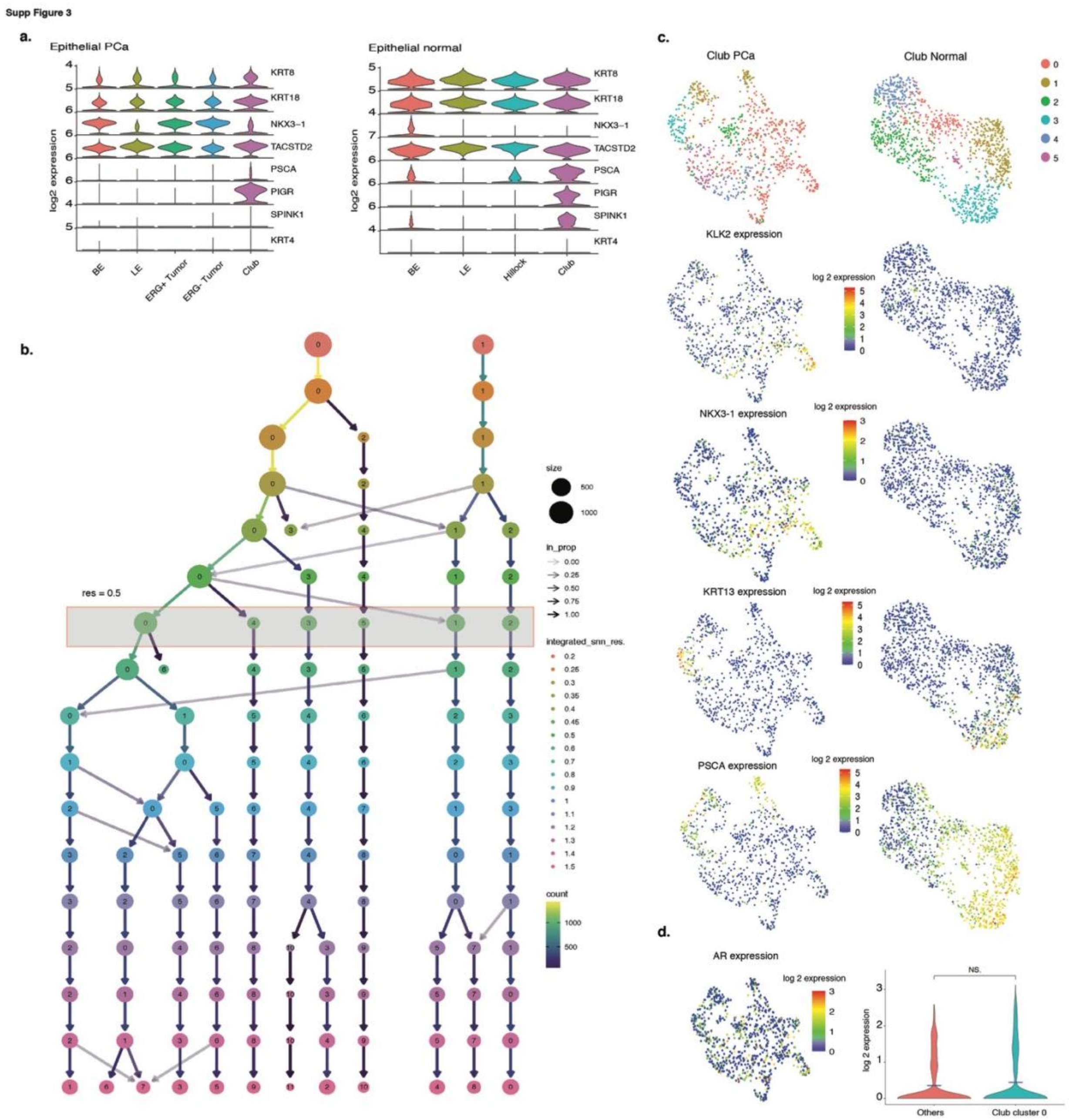

Supplementary Figure 3. Integrated club cell analysis. a. Stacked violin plots of LE progenitor cell markers for PCa (left) and normal (right) epithelial cells. **b.** Clustering stability tree for integrated club cells. Cluster transition is indicated by arrows and cluster sizes are indicated by marker sizes. Selected resolution for downstream analysis is labeled next to the highlighted region. **c.** Computed UMAP for club PCa and club

Normal. Featureplots show top expressed genes in different cell states. **d.** Comparison of *AR* expression between club cell cluster 0 and other club cells in the PCa samples (NS: p = 0.18, not significant, Wilcoxon rank sum test).

Supp Figure 4

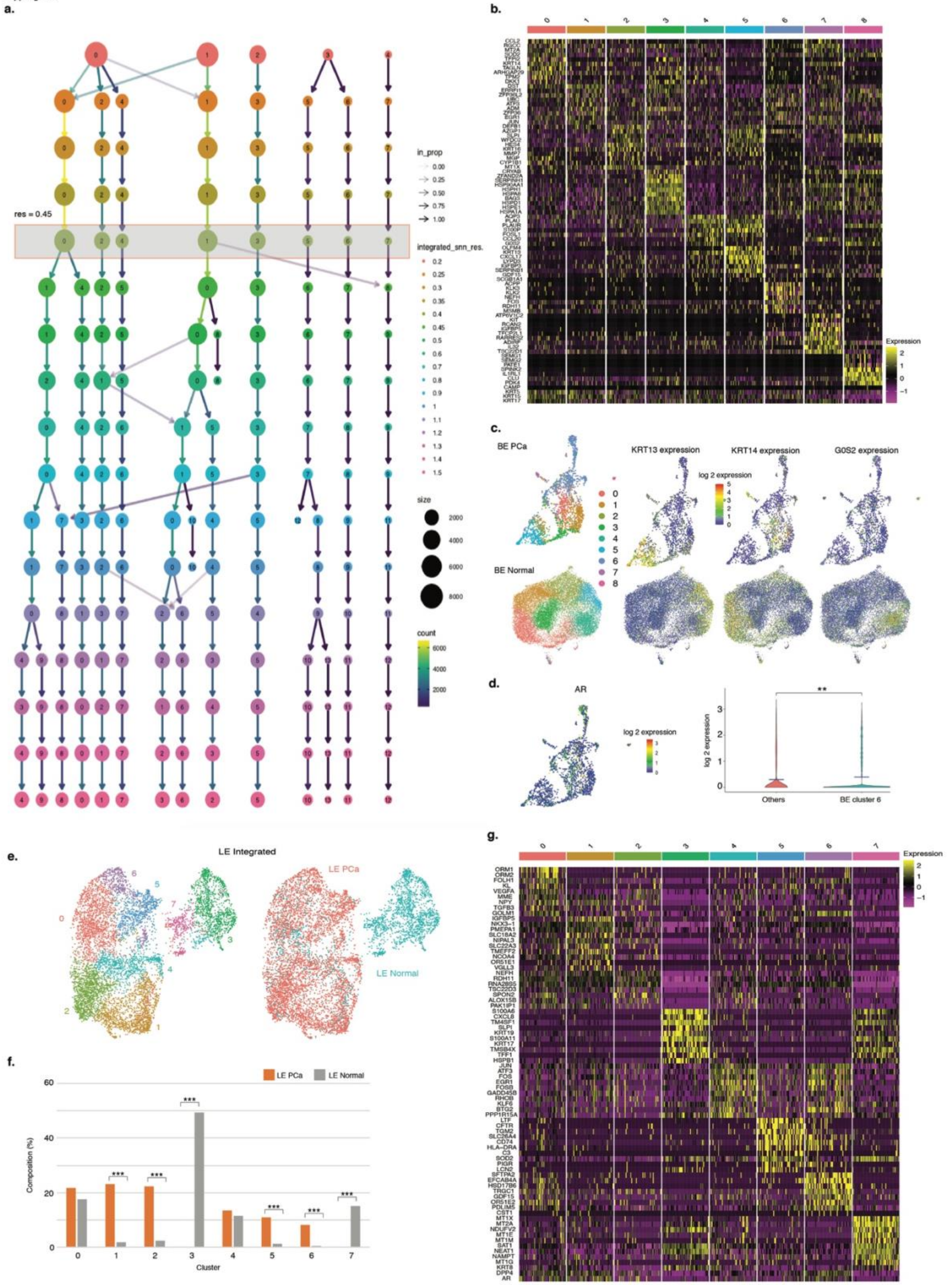

Supplementary Figure 4. Integrated BE and LE analysis. a. Clustering stability tree for integrated BE. Cluster transition is indicated by arrows and cluster sizes are indicated by marker sizes. Selected resolution for downstream analysis is labeled next to the highlighted region. **b.** Heatmap of the top 10 differentially expressed genes (DEGs) for each BE cell state. **c.** Computed UMAP for BE PCa and BE Normal. Featureplots show top expressed genes in different cell states. **d.** Comparison of *AR* expression between BE cell cluster 6 and other BE in the PCa samples (**: $p = 0.004$, Wilcoxon rank sum test). **e.** UMAP of integrated LE labeled by cell states (left) or samples type (LE PCa and LE Normal) (right). **f.** Cell composition comparison between LE PCa and LE Normal (***: $p < 0.001$, two-sided Fisher's exact test; cluster 1: p = 3.84e-152; cluster 2: p = 7.18e-132; cluster 3: p < 2.2e-16; cluster 5: p = 1.92e-60; cluster 6: p = 5.14e-56). **g.** Heatmap of the top 10 DEGs for each LE cell states. Source data are provided as a Source Data file.

Supp Figure 5

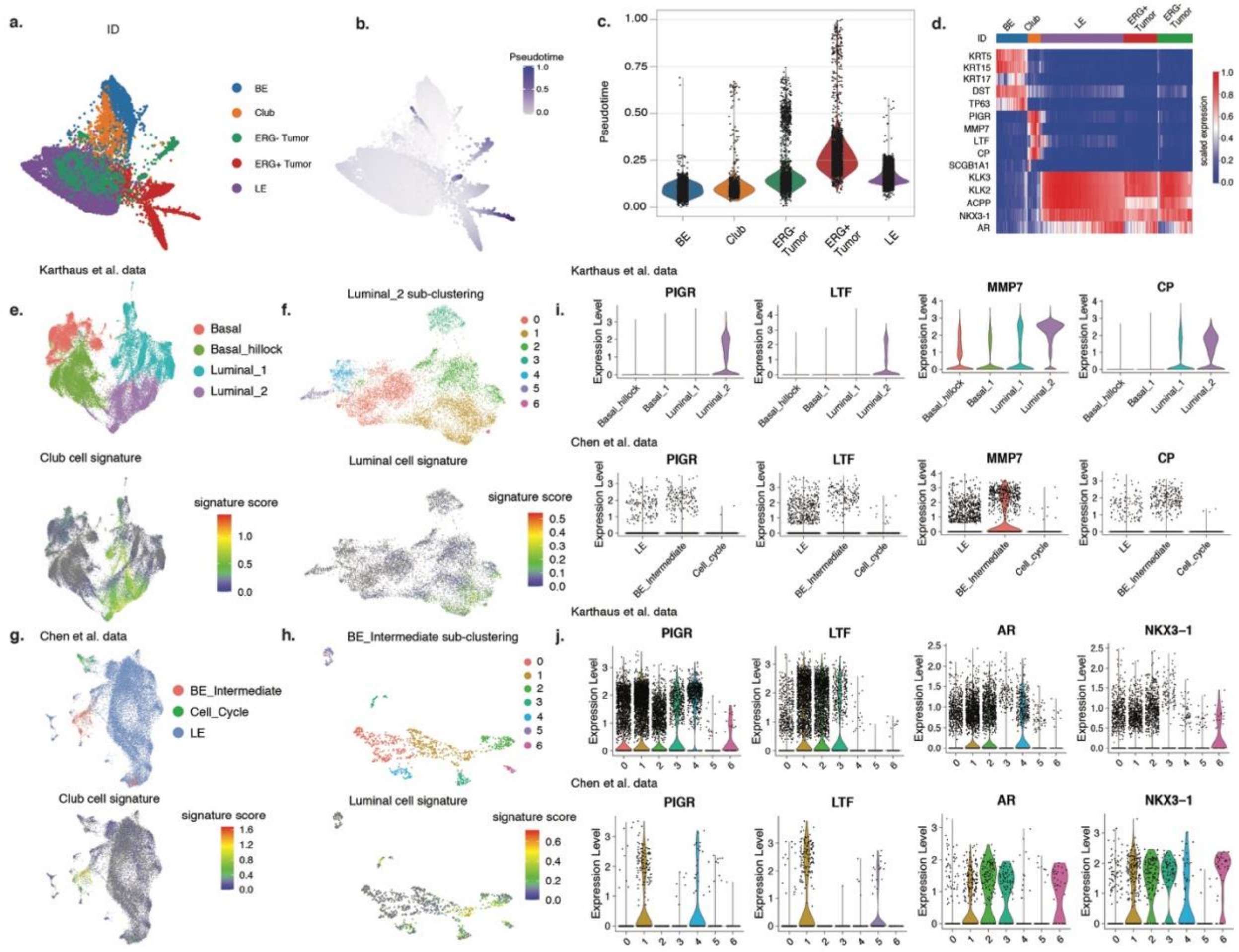

Supplementary Figure 5. Pseudotime analysis on PCa epithelial cells and club cell identification in other datasets. a. Projection of all epithelial cells in the UMAP generated by scanpy. **b.** Pseudotime trajectory of all epithelial cells. Pseudotime was illustrated by the color gradient. **c.** Violin plots of computed pseudotime by epithelial cell types. **d.** PAGA heatmap of cell type markers on all epithelial cells **e.** The UMAP of epithelial cells from the Karthaus study with the original annotation using the publicly available metadata (top) and club cell signature scores (bottom). **f.** The UMAP of epithelial cells from the Chen study with the original annotation using the publicly

available metadata (top) and club cell signature scores (bottom). **g.** Sub-clustering of the luminal-2 population in the Karthaus population (top) and club cell signature scores (bottom). **h.** Sub-clustering of previous annotated BE_Intermediate population in the Chen dataset (top) and club cell signature scores (bottom). **i.** Violin plots of PCa club cell markers *PIGR, LTF, MMP7* and *CP* for epithelial cells in the two publicly available datasets (top, Karthaus dataset; bottom, Chen dataset). **j.** Violin plots of PCa-enriched club cell state markers *PIGR, LTF, AR* and *NKX3-1* for the two sub-cluster analyses (top, Karthaus dataset; bottom, Chen dataset).

Supp Figure 6

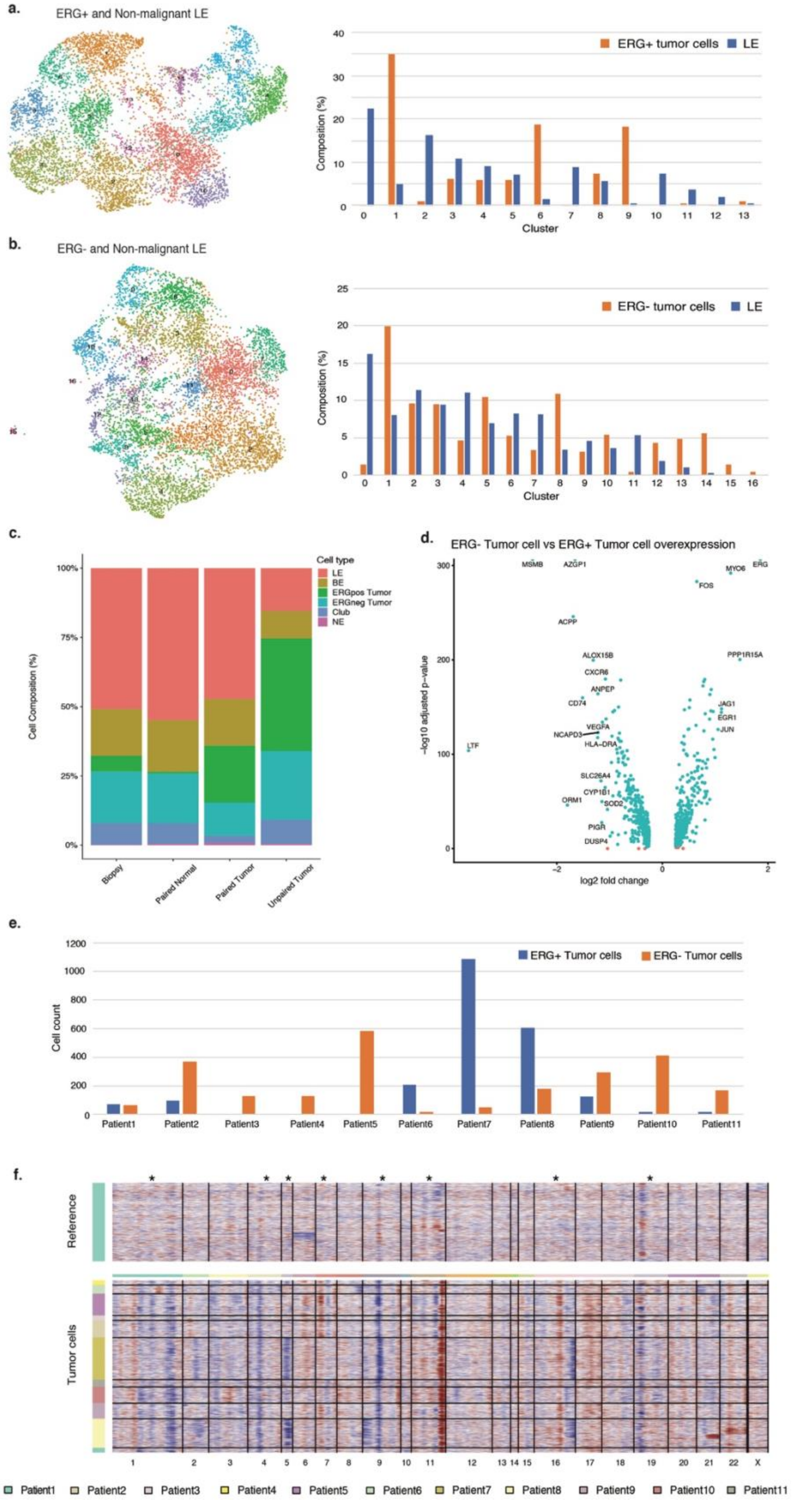

Supplementary Figure 6. Tumor cell analysis. a. Left, UMAP of integrated *ERG*-positive (*ERG*+) tumor cells and LE. Right, cluster composition comparison in grouped bar charts. **b.** Left, UMAP of integrated *ERG*- tumor cells and LE. Right, cluster composition comparison in grouped bar charts. **c.** Stacked bar chart of epithelial cells in each sample type. Composition is normalized to 100%. **d.** Volcano plots between *ERG*+ and *ERG*- tumor cells with the top 20 most overexpressed genes labeled. **e.** Group bar charts of both *ERG*+ and *ERG*- tumor cell counts in each patient. **f.** InferCNV validation of tumor cells by patients using non-tumor epithelial cells as reference. Source data are provided as a Source Data file.

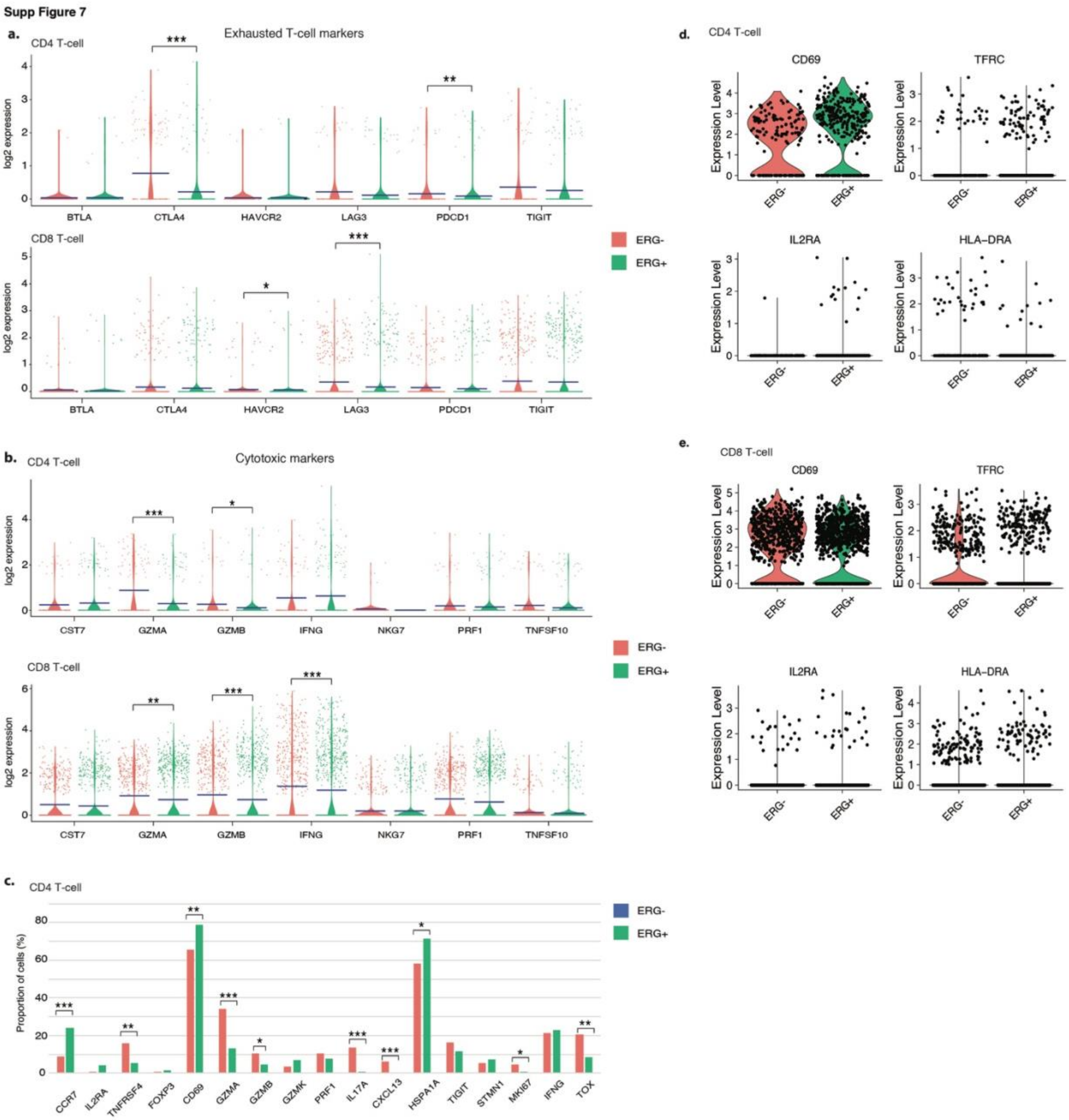

Supplementary Figure 7. ***ERG*-associated CD4 and CD8 T-cell analysis. a.** Grouped violin plots of exhausted T-cell markers for CD4 and CD8 T-cells classified by *ERG* status (***: $p < 0.001$, Wilcoxcon rank sum test; CD4 T-cells *CTLA*: $p = 6.75e\text{-}11$; *PDCD1*: $p = 0.0017$; CD8 T-cells *HAVCR2*: $p = 0.047$; *LAG3*: $p = 2.85e\text{-}08$). Mean expression level for each gene is illustrated by a solid blue line. **b.** Grouped violin plots

of cytotoxic markers for CD4 and CD8 T-cells classified by *ERG* status. Statistical significance is labeled above (*: $p < 0.05$; **: $p < 0.01$, ***: $p < 0.001$, no label: not significant, Wilcoxon rank sum test; CD4 T-cells *GZMA*: $p = 2.24e\text{-}08$; *GZMB*: $p = 0.022$; CD8 T-cells *GZMA*: $p = 0.028$; *GZMB*: $p = 2.66e\text{-}05$; *IFNG*: $p = 1.52e\text{-}04$). **c.** Frequency of expression for CD4 T-cell subtype markers between the two CD4 T-cells classified by *ERG* status (*: $q < 0.05$; **: $q < 0.01$, ***: $q < 0.001$, no label: not significant, FDR two-sided FET; *CCR7*: $q = 3.29e\text{-}4$; *TNFRSF4*: $q = 0.0018$; *CD69*: $q = 0.0099$; *GZMA*: $q = 2.49e\text{-}06$; *GZMB*: $q = 0.043$; *IL17A*: $q = 3.33e\text{-}07$; *CXCL13*: $q = 1.31e\text{-}04$; *HSPA1A*: $q = 0.015$; *MKI67*: $q = 0.011$; *TOX*: $q = 0.0012$). Mean expression level for each gene is illustrated by a solid blue line. **d.** Violin plots of T-cell activation markers for CD4 T-cells classified by *ERG* status. **e.** Violin plots of T-cell activation markers for CD8 T-cells classified by *ERG* status. Source data are provided as a Source Data file.

Supp Figure 8

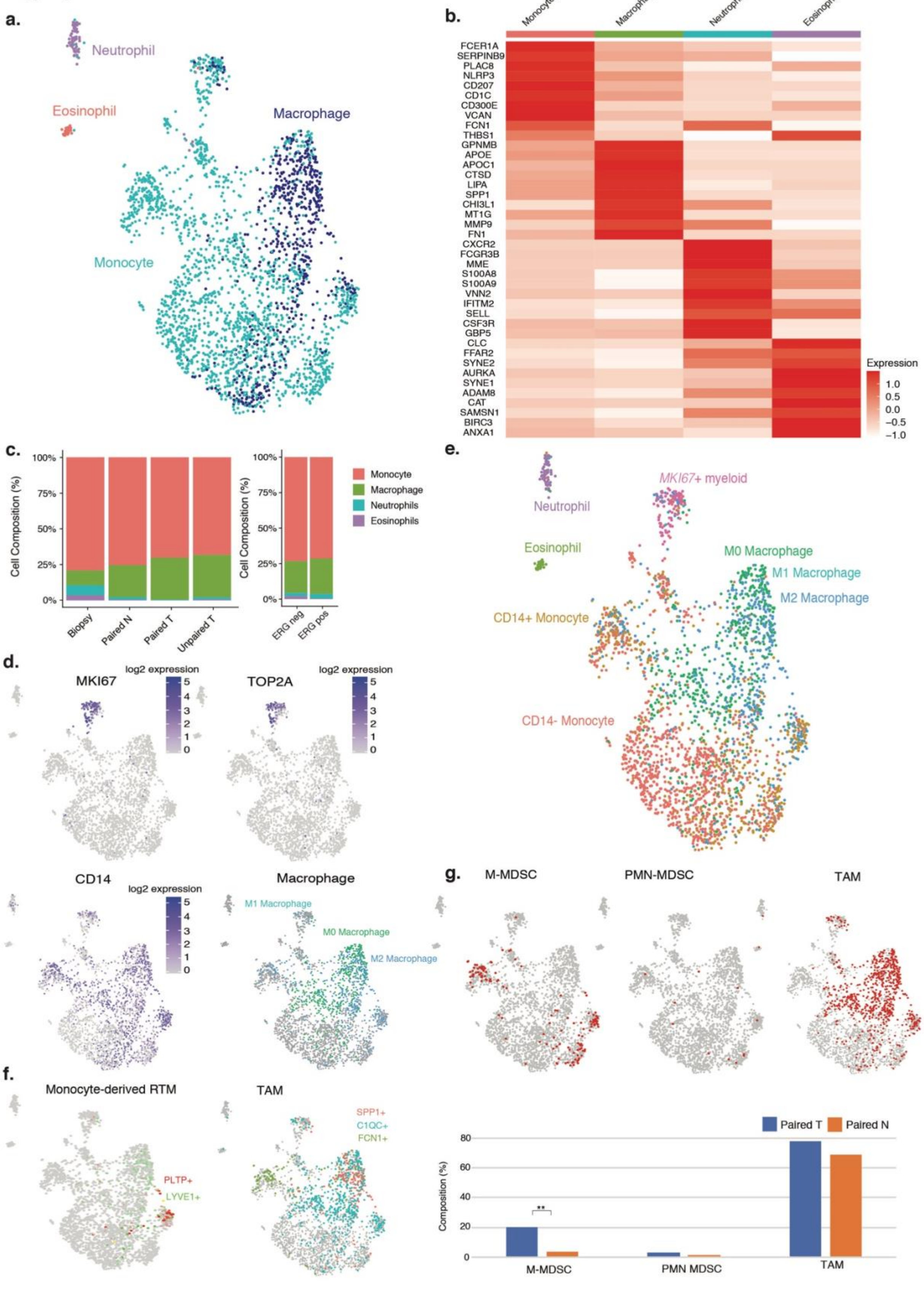

Supplementary Figure 8. Myeloid cell analysis. a. UMAP of myeloid cell population with SingleR automated annotation. **b.** Heatmap of the top 10 DEGs in each myeloid cell type. **c.** Myeloid cell composition comparison by each sample type (left) and by *ERG* status (right). **d.** Featureplots of *MKI67*, *TOP2A,* and *CD14*; distribution of M0, M1, and M2 macrophages in the myeloid cell UMAP. **e.** UMAP of myeloid cells annotated by detailed monocyte and macrophage phenotypes. **f.** Featureplots of monocyte-derived resident tissue macrophages (RTM) markers and tumor associated macrophage (TAM) markers. **g.** Top, identification of two myeloid-derived suppressor cell (MDSC) phenotypes and TAMs within myeloid cells. Bottom, composition comparison between paired tumor and paired normal samples (**: p = 0.0035, two-sided FET). Source data are provided as a Source Data file.

Supp Figure 9

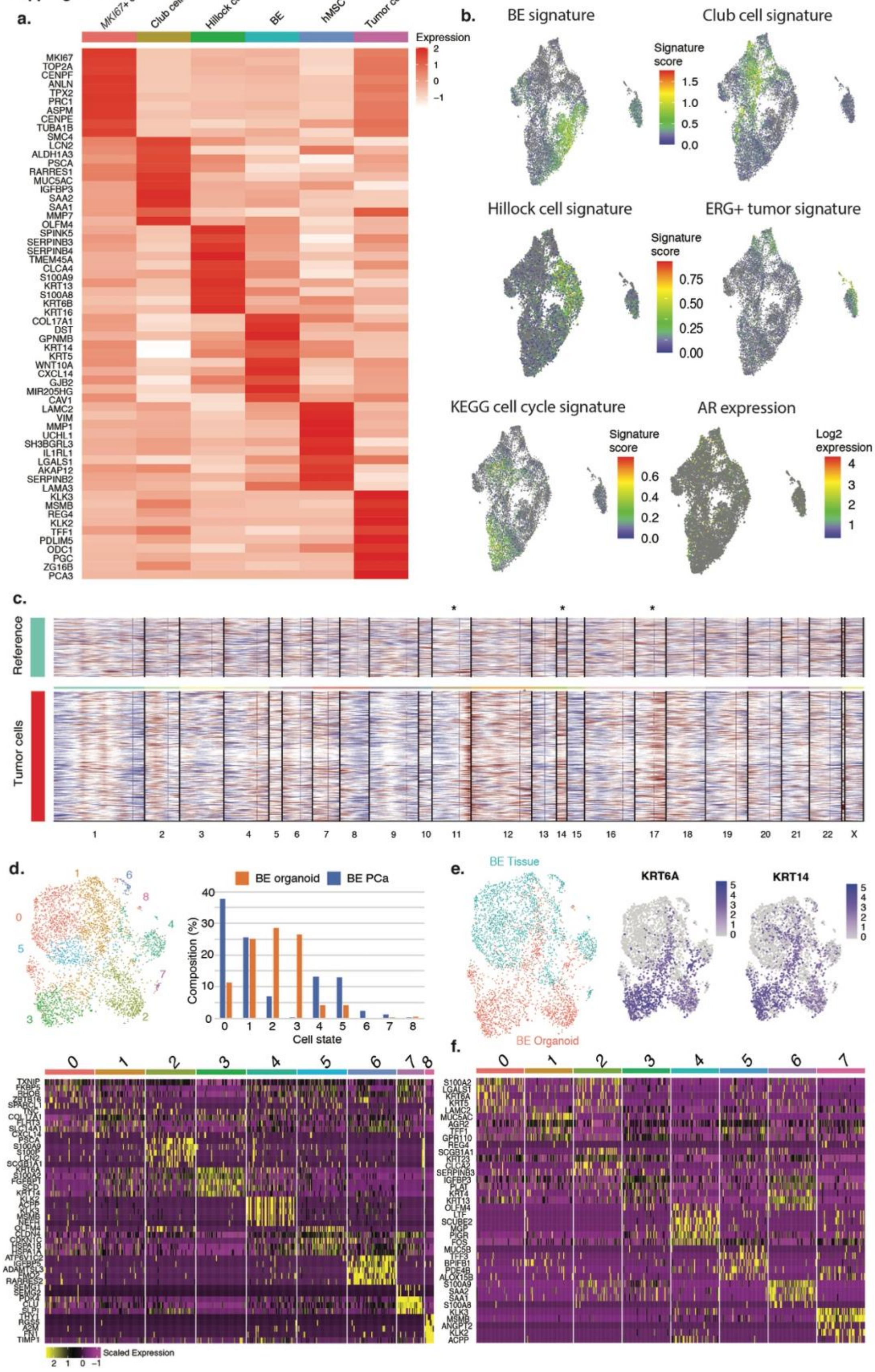

Supplementary Figure 9. Analysis on early-passage (P0-P3) organoid samples. **a.** Heatmap of the top 10 DEGs for each organoid cell type. **b.** UMAP of different tumor tissue cell type signature scores and *AR* expression Featureplot in the organoid samples. **c.** InferCNV validation using non-tumor cells as reference and tumor cells as observation. Significant copy number variations are highlighted. **d.** Top, UMAP of integrated BE dataset of tumor tissue and organoid samples and cell composition comparison grouped bar charts. Bottom, heatmap of the top 10 DEGs in the integrated BE clusters. **e.** Left, UMAP of the integrated BE cells labeled by sample type. Right, Featureplots of organoid specific BE cell state markers *KRT6A* and *KRT14*. **f.** Heatmap of the top 10 DEGs in the integrated club cell clusters. Source data are provided as a Source Data file.
