# Supplementary material for: Single-cell analysis of human primary prostate cancer reveals the heterogeneity of tumor-associated epithelial cell states
Source: Nat Commun. 2022 Jan 10;13:141. doi: 10.1038/s41467-021-27322-4 (PMC8748675; doi:10.1038/s41467-021-27322-4)
Supplement: Supplementary file 11 — Reporting Summary [file 41467_2021_27322_MOESM11_ESM.pdf]

nature portfolio

Corresponding author(s): Franklin W. Huang
Last updated by author(s): Jul 20, 2021

Reporting Summary

Nature Portfolio wishes to improve the reproducibility of the work that we publish. This form provides structure for consistency and transparency in reporting. For further information on Nature Portfolio policies, see our Editorial Policies and the Editorial Policy Checklist.

Statistics

For all statistical analyses, confirm that the following items are present in the figure legend, table legend, main text, or Methods section.

n/a	Confirmed	
☐	☒	The exact sample size (*n*) for each experimental group/condition, given as a discrete number and unit of measurement
☐	☒	A statement on whether measurements were taken from distinct samples or whether the same sample was measured repeatedly
☐	☒	The statistical test(s) used AND whether they are one- or two-sided<br>*Only common tests should be described solely by name; describe more complex techniques in the Methods section.*
☐	☒	A description of all covariates tested
☐	☒	A description of any assumptions or corrections, such as tests of normality and adjustment for multiple comparisons
☐	☒	A full description of the statistical parameters including central tendency (e.g. means) or other basic estimates (e.g. regression coefficient) AND variation (e.g. standard deviation) or associated estimates of uncertainty (e.g. confidence intervals)
☐	☒	For null hypothesis testing, the test statistic (e.g. *F*, *t*, *r*) with confidence intervals, effect sizes, degrees of freedom and *P* value noted<br>*Give P values as exact values whenever suitable.*
☒	☐	For Bayesian analysis, information on the choice of priors and Markov chain Monte Carlo settings
☒	☐	For hierarchical and complex designs, identification of the appropriate level for tests and full reporting of outcomes
☒	☐	Estimates of effect sizes (e.g. Cohen's *d*, Pearson's *r*), indicating how they were calculated

Our web collection on statistics for biologists contains articles on many of the points above.

Software and code

Policy information about availability of computer code

Data collection	Sequencing results were returned as paired FASTQ reads and processed with FastQC (v0.11.9) for general quality checks in order to further improve our experimental protocol. Then, the paired FASTQ files were aligned against the reference genome using a STAR aligner in the dropseq workflow (https://cumulus.readthedocs.io/en/latest/drop_seq.html). The aligning pipeline output included aligned and corrected bam files, two digital gene expression (DGE) matrix text files (a raw read count matrix and a UMI-collapsed read count matrix where multiple reads that matched the same UMI would be collapsed into one single UMI count) and text-file reports of basic sample qualities such as the number of beads used in the sequencing run, total number of reads, alignment logs.
Data analysis	Sequencing and Alignment<br>Sequencing results were returned as paired FASTQ reads and processed with FastQC (v0.11.9) for general quality checks in order to further improve our experimental protocol. Then, the paired FASTQ files were aligned against the reference genome using a STAR aligner (v2.7.6a) built within the dropseq workflow (Snapshot 7) (https://cumulus.readthedocs.io/en/latest/drop_seq.html). The aligning pipeline output included aligned and corrected bam files, two digital gene expression (DGE) matrix text files (a raw read count matrix and a UMI-collapsed read count matrix where multiple reads that matched the same UMI would be collapsed into one single UMI count) and text-file reports of basic sample qualities such as the number of beads used in the sequencing run, total number of reads, alignment logs. For each sample, the average number of reads was 4,875,9687, and the mean read depth per barcode was 48,586. The median and average number of genes per barcode were 767 and 1079. The median and average number of UMI were 1,335 and 2,447. The mean percentage of mitochondrial content per cell was 13.65%.<br><br>Single-cell clustering analysis<br>Cells in the samples were clustered and analyzed using customized codes based on the Seurat package (v3.2.2) in R (v4.0.3). Cells with less than 300 genes, 500 transcripts, or a mitochondrial level of 20% or greater, were filtered out as the first QC process. Then, by examining the distribution histogram of the number of genes per cell in each sample, we set the upper threshold for the number of genes per cell in each individual sample in order to filter potential doublets. A total of 22,037 cells were acquired using these thresholds. Since merging with and without integration of the samples showed no major difference in the clustering of each cell type, in the subsequent analysis of these samples

we used the merged dataset without integration.
Doublets were removed by two steps: first we used DoubletFinder (v2.0.3) and a theoretical doublet rate of 5% to locate doublets in our dataset. 305 cells marked by DoubletFinder as true positive were removed from further analysis. 21,743 cells were used in the following cell clustering analysis. Then, after clustering, we removed cells expressing biomarkers from more than one major cell type (epithelial, stromal and immune) as they were more likely to be doublets. In this step, we removed 276 cells from our dataset and the follow-up analysis, leaving 21,467 cells in total.
UMI-collapsed read counts matrices for each cell were loaded in Seurat for analysis. We followed the standard workflow by using the "LogNormalize" method that normalized the gene expression for each cell by the total expression, multiplying by a scale factor 10,000 and log-transforming the results. For downstream analysis to identify different cell types, we then calculated and returned the top 2,000 most variably expressed genes among the cells before applying a linear scaling by shifting the expression of each gene in the dataset so that the mean expression across cells was 0 and the variance was 1. This way, the gene expression level could be comparable among different cells and genes. PCA was run using the previously determined most variably expressed genes for linear dimensional reduction and the first 100 principal components (PCs) were stored which accounted for 25.42% of the total variance. To determine how many PCs to use for the clustering, a JackStraw resampling method was implemented by permutation on a subset of data (1% by default) and rerunning PCA for a total of 100 replications to select the statistically significant principle component to include for the K-nearest neighbors clustering. For graph-based clustering, the first 100 PC and a resolution of 3 were selected yielding a total of 46 cell clusters. We eliminated the clustering side effect due to overclustering by constructing a cluster tree of the average expression profile in each cluster and merging clusters together based on their positions in the cluster tree. As a result, we ensured that each cluster would have at least 10 unique differentially expressed genes (DEGs).
Differentially expressed genes in each cluster were identified using the FindAllMarker() function built within Seurat package and a corresponding p-value was given by the Wilcoxon's test followed by a Bonferroni correction. Top differentially expressed gene markers were illustrated in a stacked violin plot using a customized auxiliary function. Dot plots were generated as an alternative way of visualization using the top 10 differentially expressed genes in each cluster. Top tier cell type clustering was also validated by the automated singleR (v1.2.4) annotation (Supplemental Data 1). However, when running singleR in single-cell mode for epithelial cells, and due to the lack of detailed reference in the singleR library, singleR could not identify detailed epithelial cell types. Therefore, manual annotation was required for epithelial cells.

Cell type annotation by signature scores
In order to annotate each cell type from the previous clustering, we took the established studies and the signatures for each cell type (Supplemental Data 2). Treating the signature score of each cell type as a pseudogene, we evaluated the signature score for each cell in our dataset using the AddModuleScore() function built within Seurat. Each cluster in our dataset was assigned with an annotation of its cell type by top signature scores within the cluster.

Epithelial sub-clustering analysis and tumor cell inference
All epithelial cells were clustered using the analytical workflow described above, yielding 20 clusters. To compare the transcriptomic profiles between PCa samples and normal prostates, a previous study on normal prostate single-cell RNA-seq was downloaded and imported. Mean basal, luminal, hillock, and club signature scores were calculated for each cluster, based on the top differentially expressed genes from a previous scRNA-seq study on the normal prostate. A One-way ANOVA test was then conducted to determine if the signature score of each cluster was significantly different from the rest. We annotated the clusters with significantly upregulated basal epithelial cell (BE) signature scores as BE. Cells in clusters with high luminal epithelial (LE) signature scores could be either non-malignant luminal epithelial cells or tumor cells. The clusters with low signature scores of both BE and LE were annotated as other epithelial cells (OE). To efficiently identify tumor cells, we took the digital gene expression matrix and conducted a single set gene set enrichment analysis on GenePattern (https://gsea-msigdb.github.io/ssGSEA-gpmodule/v10/index.html) testing against the C2 gene set collection. Under the notion that tumor cells should have higher expression of one or more tumor markers overlapping existing prostate cancer gene sets, we projected the signatures of these prostate cancer gene sets on to our epithelial clusters and annotated tumor cell clusters as the clusters with significantly higher signature scores of at least one prostate cancer gene sets.
Approximately ~50% of prostate cancer cells from men of European ancestry harbor TMPRSS2-ERG fusion events, indicating high gene expression of ERG. Therefore, we hypothesized a high signature score of SETLUR PROSTATE CANCER TMPRSS2 ERG FUSION UP gene set would be a strong indicator of ERG+ tumor cells. All the other tumor cell clusters were then annotated as ERG- tumor cell clusters as they showed little to no ERG gene expression. All of the epithelial clusters with high luminal signature scores and high expression of luminal markers such as KLK3, KLK2, ACPP, KRT8 and KRT18 were annotated as non-malignant luminal epithelial cells (non-malignant LE). Compared to non-malignant cells, tumor cells harbor more single-nucleotide variants and copy number variants, leading to distinctive patterns. To validate our tumor cell annotation, we ran InferCNV (v1.4.0) on ERG+ and ERG- tumor clusters with non-malignant LEs as reference for an estimation of copy number alterations. We classified tumor cells based on ERG gene expression. Then we defined patients harboring ERG+ tumor cells as ERG+ patients and the other patients as ERG- patients. This way, we were able to classify all the other cells based on the ERG status (epithelial, stromal and immune cells) as either ERG+ or ERG-.
To determine if common functional changes were present in more than one cell type, we conducted gene set enrichment analysis (GSEA) for each cell type first and imported the significantly changed gene sets to take the intersections. Statistical significance of multi-set intersection was evaluated and visualized using the SuperExacTest package (v1.0.7).

Cell state analysis
Gene expression profile differences in epithelial cells between PCa sample and normal prostate samples were identified by integrating our PCa dataset with an established dataset on normal prostates. We utilized the integration method based on commonly-expressed anchor genes by following the Seurat integration vignette (v3.2.2) in order to remove batch effects of samples sequenced with different technologies and possible artifacts so that the cells were comparable.
In order to better characterize the transcriptomic profile and transition of cell states among identified epithelial cells, both the tumor and paired normal samples were integrated together and separately with the epithelial cells from a normal prostate scRNA-seq dataset for KRT5+ and KRT15+ basal epithelial (BE), KLK3+ and ACPP+ luminal epithelial (LE) and PIGR+ and MMP7+ club cell population together and separately. An optimal resolution value was tested using the Clustree package (v0.4.3). Heatmaps of DEGs were generated to validate the cell state differentiation. Compositions for each cell state was computed and compared between PCa samples and normal samples using Fisher's exact test.
To assess the functional roles of the PCa-enriched cell states identified within the integrated dataset, we ran GSEA analysis between the PCa-enriched cell state and all the other cell states as a whole. The top 20 downregulated and upregulated gene sets were visualized in terms of gene counts and ratio for each gene set. Using the DEGs from each cell state, we generated signature gene sets for all the cell states in BE, LE and club cells. To validate the functional implications for the PCa-enriched cell states, we conducted single set gene set enrichment analysis (ssGSEA) on PCa BE and club cells to compute the signature scores of the upregulated gene sets using the ssGSEA module on GenePattern (https://gsea-msigdb.github.io/ssGSEA-gpmodule/v10/index.html). Then, we computed the information coefficient (IC) and corresponding p-

values followed by FDR correction to evaluate the correlation between these gene sets and cell states.

Pseudotime analysis
To evaluate the epithelial cell states with respect to their order in the differentiation trajectory, we conducted pseudotime analysis on all epithelial and tumor cells identified in the PCa samples. We first calculated a PAGA (partition-based graph abstraction) graph using SCANPY's sc.tl.paga() function (v1.8.1) and then used sc.tl.draw_graph() to generate the PAGA initialized single-cell embedding of the cell types. The diffusion pseudotime for each cell was calculated using SCANPY's sc.tl.diffmap() and sc.tl.dpt() with the root cluster as the BE cluster and then was plotted on the PAGA initialized embedding. We then visualized the gene marker changes along the pseudotime by cell type using sc.pl.paga_path().

scRNA-seq Fusion detection
Fusion transcripts were detected using STAR-Fusion (v1.6.0). STAR-Fusion was run from a Docker container using the following options: --FusionInspectorvalidate, --examine_coding_effect, and –denovo_reconstruct. Due to the low coverage of scRNA-seq samples, both filtered fusion detection results and preliminary results were combined and processed, in which we only filtered for potential TMPRSS2-ERG fusion events.

Bulk RNA-sequencing validation
Two publicly available bulk RNA-sequencing PCa datasets were used to test the correlation between the PCa-enriched cell state signatures and AR signaling, including Prostate Adenocarcinoma (TCGA, Firehose Legacy) dataset and Metastatic Prostate Cancer, SU2C/PCF Dream Team (SU2C49, PNAS 2019) dataset. For each dataset, mRNA expression was downloaded and normalized. Signature scores of AR signaling (Hallmark androgen response pathway), BE, LE and club cell states as well as ERG+ and ERG- tumor cell signature scores were computed for each sample via ssGSEA analysis. Samples in each dataset were rank ordered by the AR signature scores and heatmaps were generated using the customized scripts. To test the correlation between AR signature scores and each cell state signature score, we computed the information coefficient and corresponding p-values followed by FDR correction to evaluate the correlation. For tumor cell signatures, we computed the correlations between the ERG fusion status from each dataset and the signature scores of ERG+ and ERG- tumor cell gene sets we had previously generated. We ranked ordered the bulk RNA-seq samples according to whether or not the TMPRSS2-ERG fusion was detected and plotted the ERG+ and ERG- tumor cell signature score heatmaps. Information coefficient (IC), p-values and FDR q-values were computed.

Immune cell analysis
T-cell and myeloid cell populations were sub-clustered separately following a similar pipeline as described above. For T-cells, 23 PCs and a resolution of 1.5 were selected for the clustering. For myeloid cells, 27 PCs and a resolution of 1.5 were selected. Cell clusters were annotated by a dot plot showing the top 10 most expressed genes in each cluster.
Monocytes, macrophages, neutrophils and eosinophils were identified and annotated based on the automated SingleR analysis. M1, M2 macrophage phenotypes, tumor associated macrophages and two types of myeloid-derived suppressor cells were identified using documented markers from previous studies.

All relevant datasets in this study are reported in the data availability section of the manuscript.

For manuscripts utilizing custom algorithms or software that are central to the research but not yet described in published literature, software must be made available to editors and reviewers. We strongly encourage code deposition in a community repository (e.g. GitHub). See the Nature Portfolio guidelines for submitting code & software for further information.

Data

Policy information about availability of data

All manuscripts must include a data availability statement. This statement should provide the following information, where applicable:

- Accession codes, unique identifiers, or web links for publicly available datasets
- A description of any restrictions on data availability
- For clinical datasets or third party data, please ensure that the statement adheres to our policy

All relevant datasets in this study are reported in the data availability section of the manuscript.

Field-specific reporting

Please select the one below that is the best fit for your research. If you are not sure, read the appropriate sections before making your selection.

☒ Life sciences ☐ Behavioural & social sciences ☐ Ecological, evolutionary & environmental sciences

For a reference copy of the document with all sections, see nature.com/documents/nr-reporting-summary-flat.pdf

Life sciences study design

All studies must disclose on these points even when the disclosure is negative.

Sample size

11 localized prostate cancer patients were enrolled in this study. We obtained 6 prostate biopsies from 3 different patients (2 biopsies each for patient 1-3, obtained at the same time point), 4 radical prostatectomies (RP) with tumor-only samples from 4 patients (patients 4-7) and 4 radical prostatectomies with matched normal samples from 4 patients (patients 8-11). All RP patients had palpable lesions that were visible on preoperative MRI or ultrasound and were later confirmed pathologically to be cancer. Matched normal samples were taken from palpably normal regions). Of these 11 patients, only one patient (patient 4) was treated with finasteride. A total of 21,743 cells were analyzed in this study.

Data exclusions	QC was conducted in our analytical pipeline to remove low-quality cells. Cells with less than 300 genes, 500 transcripts, or a mitochondrial level of 20% or greater
Replication	Processed single-cell RNA sequencing data needed for reproducibility of our analytical findings are deposited in the NCBI GEO database to ensure replication of the analysis. Technical and biological replicates reproduced the shown staining. Due to the limitation of organoid sizes and subsequently image quality, we only showed one group of experimental results in Figure 8b. We included the reproducibility statements in the legends of Figure 8 and Supplementary Figure 2.
Randomization	Samples were not allocated into experimental groups.
Blinding	Blinding was not relevant to this study.

Reporting for specific materials, systems and methods

We require information from authors about some types of materials, experimental systems and methods used in many studies. Here, indicate whether each material, system or method listed is relevant to your study. If you are not sure if a list item applies to your research, read the appropriate section before selecting a response.

Materials & experimental systems

n/a	Involved in the study	
☐	☒	Antibodies
☒	☐	Eukaryotic cell lines
☒	☐	Palaeontology and archaeology
☒	☐	Animals and other organisms
☐	☒	Human research participants
☒	☐	Clinical data
☒	☐	Dual use research of concern

Methods

n/a	Involved in the study	
☒	☐	ChIP-seq
☒	☐	Flow cytometry
☒	☐	MRI-based neuroimaging

Antibodies

Antibodies used	Alexa Fluor 488-AffiniPure Donkey Anti-Chicken IgY (IgG) (H+L) (Jackson ImmunoResearch, Cat: 703-545-155, 1:500),<br>Donkey anti-Mouse IgG (H+L) Cross-Adsorbed Secondary Antibody, DyLight 550 (Thermo Fisher Scientific, Cat: SA5-10167, 1:500),<br>Donkey anti-Rat IgG (H+L) Cross-Adsorbed Secondary Antibody, DyLight 680 (Thermo Fisher Scientific, Cat: SA5-10030, 1:500),<br>Alexa Fluor 790 AffiniPure Donkey Anti-Guinea Pig IgG (H+L) (Jackson ImmunoResearch, Cat: 706-655-148, 1:500) containing DAPI (Sigma, Cat: D9542-5MG, 1:1000),<br>monoclonal mouse anti-Lactoferrin (Abcam, Cat: ab10110, 1ug/mL),<br>monoclonal rat anti-Uteroglobin/SCGB1A1 (R&D Systems, Cat: MAB4218-SP, 1ug/mL),<br>polyclonal guinea pig anti-Cytokeratin 8 + 18 (Fitzgerald, Cat: 20R-CP004, 1:100),<br>polyclonal chicken anti-Keratin 5 (Biolegend, Cat: 905901, 1:100).
Validation	All antibodies used in this study were obtained from commercial source, and validated according to manufacturers' instruction.

Human research participants

Policy information about studies involving human research participants

Population characteristics	Population characteristics are outlined in Supplementary Table 1 with patient identifiers removed.<br>We collected samples from primary prostate cancer patients.<br>Other characteristics were not used as covariates in the study.
Recruitment	11 localized prostate cancer patients who presented for urologic care were enrolled in this study. We obtained 6 prostate biopsies from 3 different patients (2 biopsies each for patient 1-3, obtained at the same time point), 4 radical prostatectomies (RP) with tumor-only samples from 4 patients (patients 4-7) and 4 radical prostatectomies with tumor and matched normal samples from 4 patients (patients 8-11). All RP patients had lesions that were visible on preoperative MRI or ultrasound and were later confirmed pathologically to be cancer.
Ethics oversight	We included the following statement in the manuscript. The UCSF Institutional Review Board (IRB) committee approved the collection of these patient data included in this study.

Note that full information on the approval of the study protocol must also be provided in the manuscript.
